# The c-jun N-terminal kinase plays a key role in ocular degenerative changes in a mouse model of Alzheimer disease suggesting a correlation between ocular and brain pathologies

**DOI:** 10.18632/oncotarget.19886

**Published:** 2017-08-03

**Authors:** Lucia Buccarello, Alessandra Sclip, Matteo Sacchi, Anna Maria Castaldo, Ilaria Bertani, Andrea ReCecconi, Silvia Maestroni, Gianpaolo Zerbini, Paolo Nucci, Tiziana Borsello

**Affiliations:** ^1^ IRCCS-Istituto di Ricerche Farmacologiche “Mario Negri”, Milan, Italy; ^2^ Department of Pharmacological and Biomolecular Sciences, University of Milan, Milan, Italy; ^3^ University Eye Clinic, San Giuseppe Hospital, University of Milan, Milan, Italy; ^4^ Unità Complicanze del Diabete, Istituto Scientifico San Raffaele, Milan, Italy

**Keywords:** Alzheimer’s disease, stress signaling pathway, JNK, retinal ganglion cell layer, optical coherence tomography

## Abstract

Recently a range of ocular manifestations such as retinal and lens amyloid-beta accumulation and retinal nerve fiber layer loss have been proposed as potential biomarkers in Alzheimer disease (AD). The TgCRND8 mouse model of AD exhibits age-dependent amyloid β (Aβ) oligomers accumulation and cognitive defects, amyloid plaques and hyperphosphorylated Tau deposition and inflammation. We proved the correlation between ocular pathologies and AD, observing increased levels of p-APP and p-Tau, accumulation of Aβ oligomers in the retina, eye, and optic nerve. The accumulation of amyloid markers was significantly stronger in the retinal ganglion cell (RGC) layer, suggesting that RGC might be more susceptible to degeneration. We detected a thinning of the RGC layer as well as RGC death in the retina of TgCRND8 mice, by using a combination of Optical Coherence Tomography (OCT), immunofluorescence, immunohistochemistry and Western blotting techniques. We proved for the first time the key role of C-Jun N-terminal Kinase (JNK) in the ocular degeneration. In support of this, the administration of the JNK inhibitor, D-JNKI1, was able to counteract the Aβ and p-Tau accumulation in the retina of TgCRND8 mice, and consequently reduce RGCs loss. These results confirm that degenerative changes in the retina/eye of AD mouse model mirrors the events observed in the brain parenchyma. Ocular changes can be detected by non-invasive imaging techniques, such as OCT, to study and test different therapeutic strategies against degenerative events associated to AD.

## INTRODUCTION

Alzheimer Disease (AD) is a chronic neurode-generative disease that affects 20-40% of people older than 85 years with a relevant social and economic impact on society [1]. AD is characterized by the accumulation of protein aggregates in the brain parenchyma, leading to synaptic dysfunction, chronic inflammation and cognitive impairments [2-3]. Extracellular senile plaques are formed by aggregation of Aβ peptides, produced by the processing of the amyloid precursor protein (APP), while intracellular accumulation of Tau protein leads to the formation of neurofibrillary tangles (NFTs). Recently, accumulation of Aβ plaques and neurofibrillary tangles has been observed in the retina as well as in visual areas of the brain of AD patients [4]. Moreover, AD patients present neuropathological alterations of the visual system and changes within ocular structures [5-7] at early stages of the disease. Interestingly, some visual pathological symptoms can precede the onset of dementia [8-9]; therefore, ocular scan could be used as a potential tool for AD diagnosis and also for monitoring AD progression [10-11].

The optical coherence tomography (OCT) is an *in vivo* non-invasive technique that provides cross-section imaging of the retina and allows to measure the retinal nerve fibers layer (RNFL). Using OCT, it has been shown that AD patients present significant thinning of the RNFL [12-15]. Currently, the most accredited hypothesis for ocular changes related to AD pathology suggests that the retinal ganglion cells (RGCs) die following accumulation of Aβ toxic species in the retina [5, 16-17], therefore leading to the thinning of the RNFL. To support this hypothesis, it has been shown that intra-vitreal injection of Aβ oligomers causes RGC damage [18-19].

In addition, several studies have described the association between AD and glaucoma. In fact, retinal disorders such as glaucoma are chronic neurodegenerative conditions, that affect retinal neurons leading to progressive and irreversible loss of vision in patients with AD [18, 20-21]. The manifestation of glaucoma is associated to an increase of intraocular pressure and an irreversible RGCs loss leading to a damage to the optic nerve [22]. Although glaucoma is not defined as an amyloidogenic disease, recently many animal and human studies highlighted the progressive accumulation of Aβ fragments in the retina [23] and its correlation to the increased intraocular pressure and RGCs apoptosis [23].

For these reasons, there is an expanding interest in the evaluation of the retina as a mirror of the CNS, particularly as a model to study AD pathology. In fact, a greater understanding of the link between ocular disorders and AD disease may help in the identification of some overlapping molecular mechanisms this will help in the development of novel therapeutic strategies, which are missing to date.

In this study we used a well-characterized murine model of AD, the TgCRND8 mouse model, to study retina/ocular pathological changes and ocular biomarkers for early detection of AD. We previously showed that TgCRND8 mice develop an early cognitive impairment, at 3 months of age, due to increasing Aβ oligomers production followed by phosphorylation of APP, as well as p-Tau deposition and inflammation [24-26]. Moreover, in these mice we showed that the JNK signaling pathway plays a key role in the pathogenesis of AD, and that the inhibition of JNK through D-JNKI1 exerts a protective effect if administrated at early and late stages of the disease [24-25].

To verify if JNK is implicated also in ocular degeneration, we focusing on RGCs death and ocular AD related-changes such as: Aβ oligomers accumulation, APP and Tau alterations, searching similar molecular mechanisms previously found in brain parenchyma [24]. Secondly, we analyzed the effect of D-JNKI1 against ocular pathological process in TgCRND8 mice.

We demonstrated that TgCRND8 mice present a significant thinning of the RNFL layers and a reduction in the number of RGCs in the retina. Moreover, we found in both the retina as well as the total eye homogenates an accumulation of Aβ _1-42_ toxic species, p-APP and p-Tau, well-recapitulating the neuropathological changes observed in hippocampal and cortical brain regions [24]. In addition, TgCRND8 mice showed a powerful activation of the JNK signaling pathway in the retina, as previously demonstrated in hippocampal and cortical tissues, corroborating the hypothesis that the retina and the brain shared similar intracellular degenerative signaling pathways. Finally, D-JNKI1 treatment prevented AD-related alterations in eye-homogenates and retinas, preserving the number of RGC and the thickness of the RNFL layer. These results indicate that eye represents an important window of the brain and can be used to monitor AD progression as well as efficacy of new treatments, being easily accessible with non-invasive optical imaging techniques as OCT.

## RESULTS

### TgCRND8 mice present accumulation of toxic Aβ species and tau in the retina as well as activation of the JNK signaling pathway

**Figure 1:**
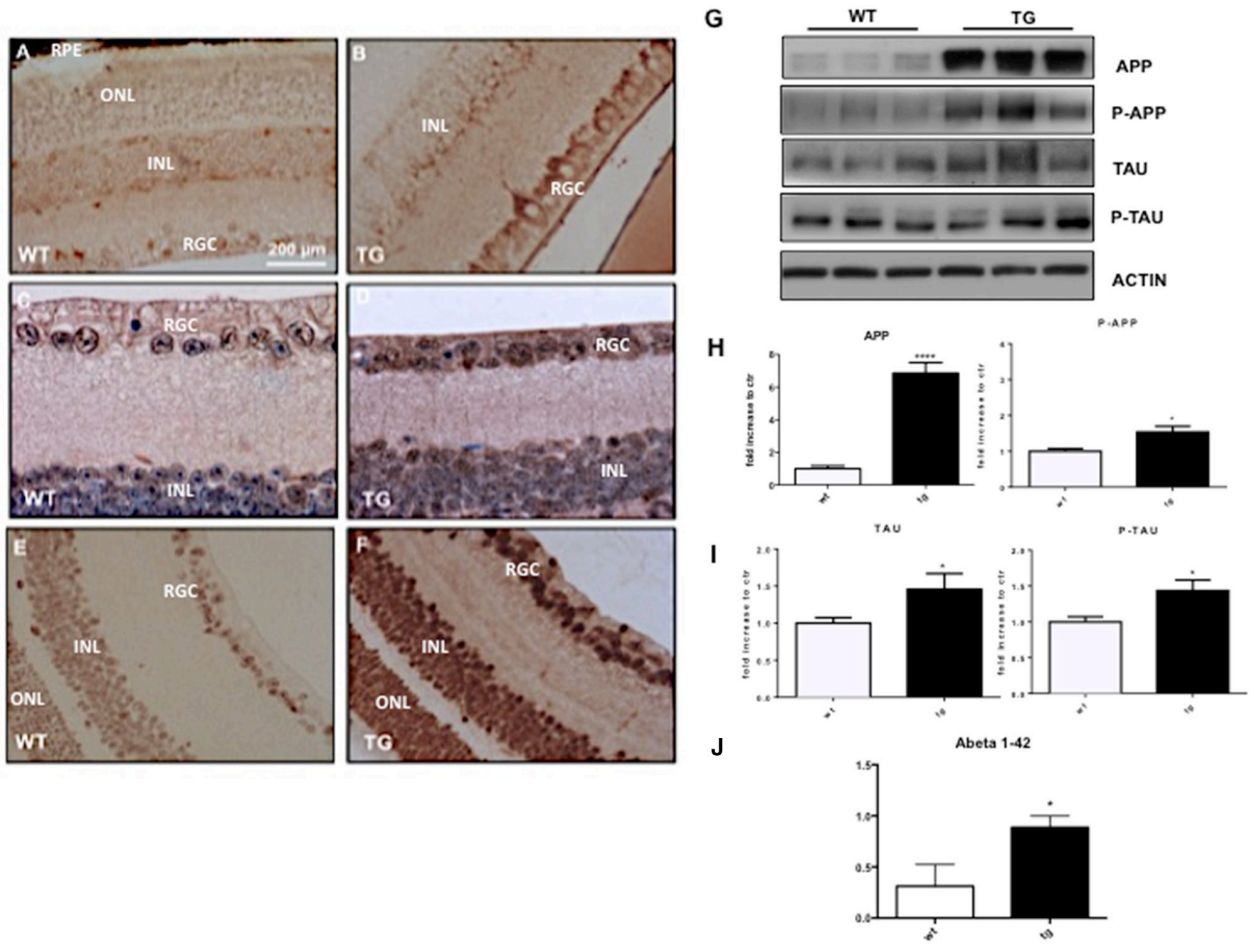
Deposition and quantification of Aβ plaques, APP and hyperphosphorylated Tau in the retina of TgCRND8 mice Retinal flat mount indicating deposition of APP **(A-B)**, 6e10 **(C-D)** positive amyloid aggregates, and P-Tau **(E-F)** in TgCRND8 mice [left] and WT mice [right] [Scale 200 μm]. **(G-I)** Western blot analysis showed altered level of APP/P-APP, Tau/P-Tau [bands highlighted] in retinal lysates from Tg compared to WT animals and their densitometric quantification. **(J)** Quantitative determination of beta amyloid fragments [1-42] in the retina by ELISA assay, showing an increased in the level of Aβ 1–42 in TgCRND8 compared to control mice. Data are expressed as mean ±SEM, Student’s T-test *p< 0.05. Data are expressed as mean ±SEM. Student’s t-test: *p< 0.05, *** p< 0.001; n=6.

To detect typical hallmarks of AD, such as amyloid plaques and hyperphosphorylated Tau, we performed immunohistochemical analysis on the retina of TgCRND8 (tg) and wild-type mice (WT). We observed an over-expression of APP and Aβ oligomers in the retina of TgCRND8 by using respectively an antibody against APP and the 6E10 antibody to detect the beta amyloid deposition (Figure 1A-1D). We described for the first time a significant increase in the immuno-reactivity of APP in the Retinal Ganglion Cells (RGC layer), as well as a less marked APP staining in the inner nuclear layer (INL) (Figure 1A-1B) of 4-month-old TgCRND8 compared to wild type mice. Using the 6E10 antibody, we observed a similar staining pattern (Figure 1C-1D). We then evaluated aggregates of hyperphosphorylated Tau (p-Tau) in the retina of TgCRND8 mice, observing for the first time a strongly increase of immune-positive staining of p-Tau compared to WT mice (Figure 1E-1F). The p-Tau immunoreactivity was strong in the RGC layer, and detectable at lower levels in the outer nuclear layer (ONL) and in the retinal pigment epithelium (RPE) (Figure 1E-1F). The evaluation of APP, p-APP, Tau and p-Tau levels was additionally performed and quantified by Western blotting analysis. We found that the level of APP was increased by 5 folds in the retina of TgCRND8 mice compared to WT mice (Figure 1G-1H), while the p-APP level was significantly increased by 1.5 folds (Figure 1G-1H, see quantification). In a similar manner, Tau and p-Tau were augmented by 1.5 folds in TgCRND8 mice compared to wild type mice (Figure 1G, I see densitometric quantification). Moreover, we also detected Aβ toxic species in retina homogenates from TgCRND8 mice, using the ELISA assay, underlyng a significant increase of Aβ oligomers formation in tg mice compared to WT mice (Figure 1J).

**Figure 2:**
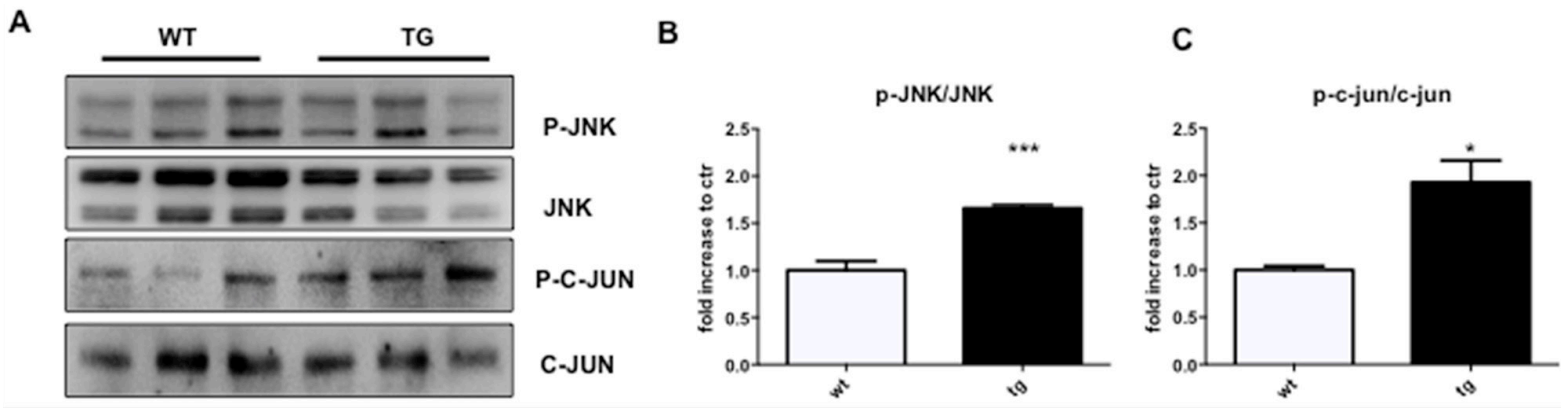
Detection of JNK signalling in the retina of TgCRND8 mice **(A)** Western blot and **(B-C)** relative quantification showing P-JNK, JNK, P-c-Jun and c-Jun in retinal lysates from 4-month-old WT and TgCRND8 mice. P-JNK/JNK ratio and P-c-Jun/c-Jun ratio were increased in TgCRND8 mice if compared with the age-matched WT mice. Data are expressed as mean ±SEM. Student’s t-test, P-JNK/JNK: *** p< 0.001; P-c-Jun/c-Jun: *p< 0.05 [n=6].

Since we proved that c-Jun N-terminal kinase (JNK) played a pivotal role in AD pathogenesis, being activated in cortical and hippocampal neurons at early stages of the disease [24-25, 27], we here investigated if similar activation of the JNK pathway was observed in the retina of TgCRND8 mice. We found that the p-JNK/JNK ratio was significantly increased by 1.7 folds in retina homogenates from TgCRND8 vs WT mice (Figure 2A-2B). To confirm the activation of the JNK pathway in TgCRND8 mice, we also measure the level of P-c-Jun/c-Jun, the elective target of JNK. As shown in Figure 2, the P-c-Jun/c-Jun ratio was increased by 2-folds in the retina of TgCRND8 mice compared to WT mice (Figure 2A-2C).

### AD hallmarks can be detected in whole eye homogenate from TgCRND8

**Figure 3:**
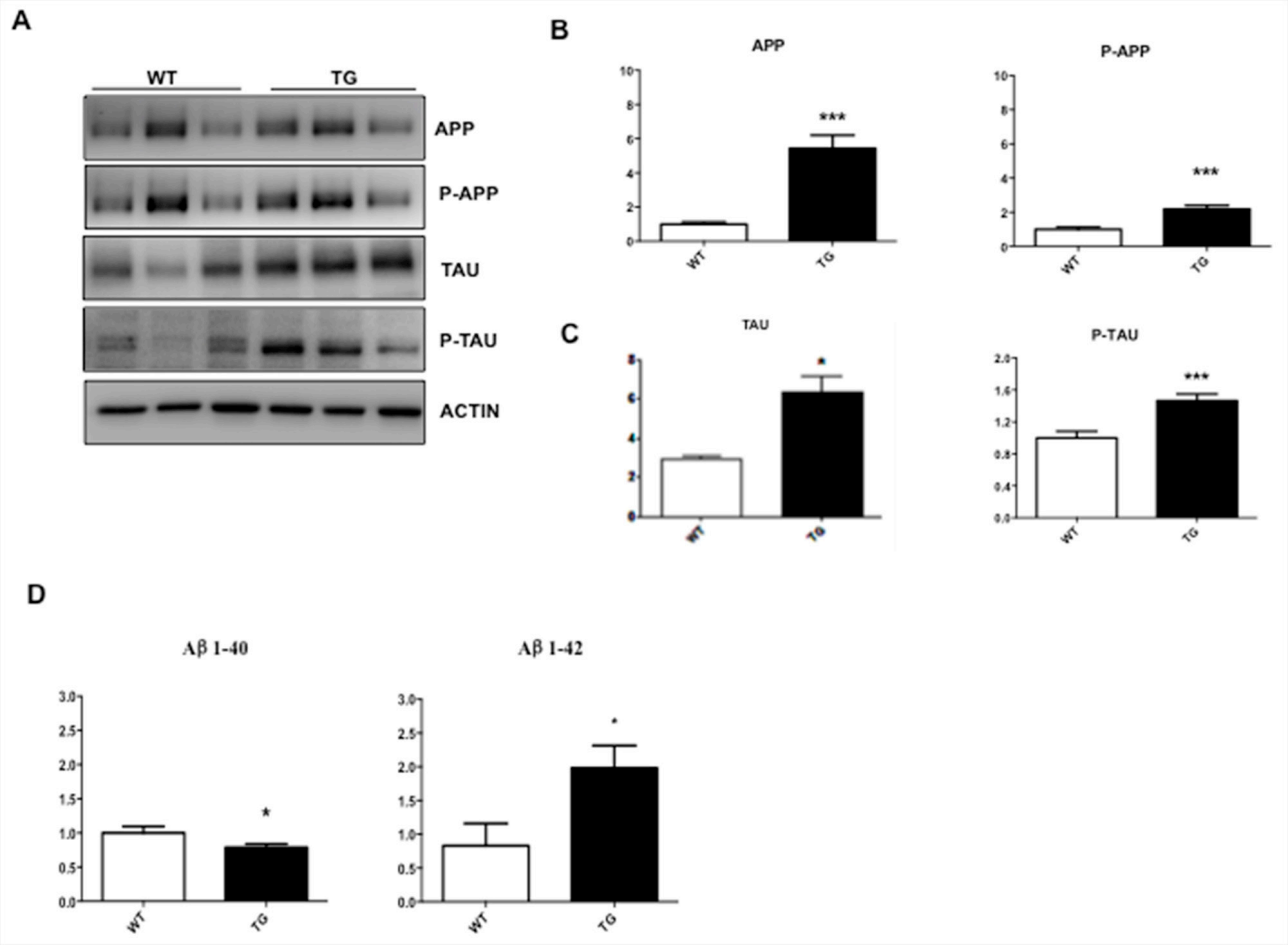
Characterization of whole eye extracts in TgCRND8 mice **(A)** Western blot analysis and **(B-C)** their densitometric quantification showed an increase of APP, P-APP, Tau, P-Tau aggregation in whole eye extracts from Tg compared to WT animals. **(D)** Quantitative determination of beta amyloid fragments [1-40 and 1-42] in whole eye extracts by ELISA assay. A decrease in the level of Aβ 1–40 fragments and an increase in the level of toxic Aβ 1–42 fragments was observed in TgCRND8 compared to control mice. Student’s t-test, *p< 0.05. Data are expressed as mean ±SEM. Student’s t-test: *p< 0.05, *** p< 0.001 [n=6].

**Figure 4:**
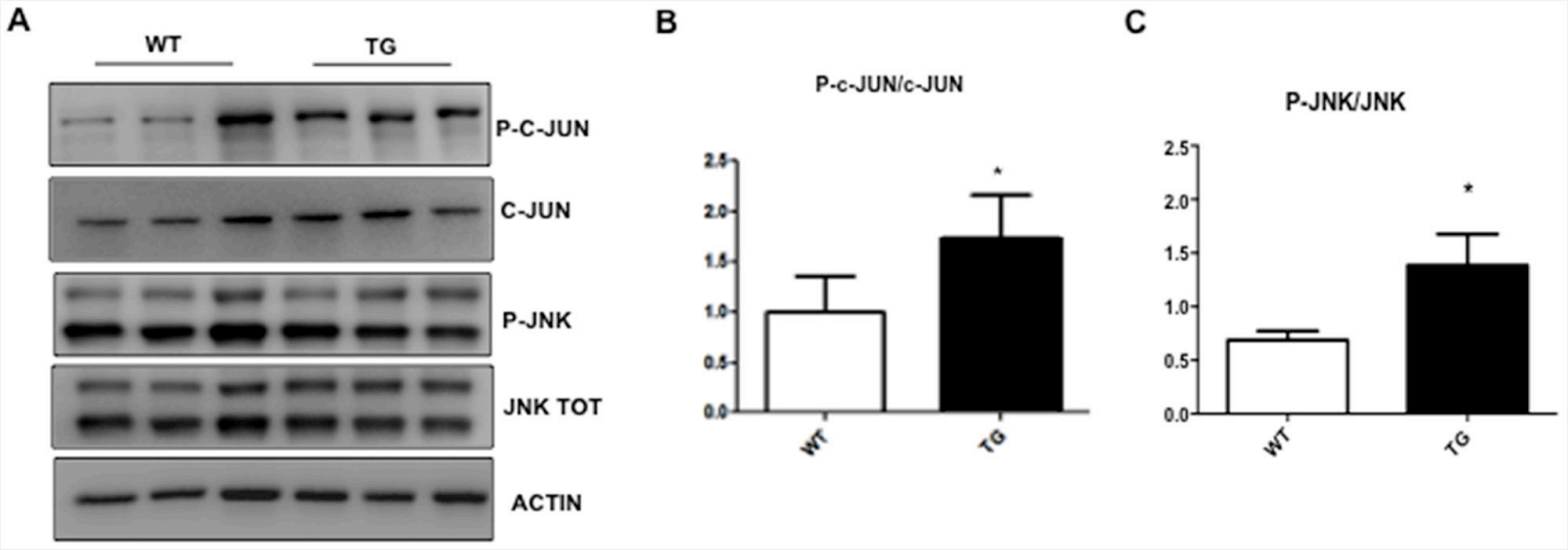
Characterization of JNK pathway in eye whole extract of TgCRND8 mice **(A)** Western blot and **(B-C)** relative quantification showing P-JNK, JNK, P-c-Jun and c-Jun in whole extract of eye from 4-month-old WT and TgCRND8 mice. P-c-Jun/c-Jun and P-JNK/JNK ratio were increased in TgCRND8 mice if compared with the age-matched WT mice. Data are expressed as mean ±SEM. Student’s t-test, *p< 0.05, n=6.

We investigated if changes detected in the retina of TgCRND8 mice could be also observed in whole eye homogenates, which are easier to handle in mice. As previously showed in the retina (Figure 1), in whole eye homogenates, the APP level increased by 5 folds in TgCRND8 compared to WT (p< 0.0001, Figure 3A-3B), while p-APP increased by 2 folds (Figure 3A-3B). Similarly, Tau levels increased by 5 folds in TgCRND8 (p< 0.05, Figure 3A-3C), while p-Tau was double in TgCRND8 mice compare to WT (p< 0.0001, Figure 3A-C). Coherently with the increased level of APP and Tau and their phosphorylated forms, the ELISA kit revealed an increase in the level of Aβ _1-42_ toxic species and a relative decreased of Aβ_1-40_ species in TgCRND8 if compared to WT total eye homogenates (p< 0.05, Figure 3, 3D). In the whole eye homogenate, the p-JNK/JNK as well as P-c-Jun/c-Jun ratios were double in TgCRND8 compared to WT (p< 0.05, Figure 4A-4C), confirming a powerful activation of the JNK signalling pathway.

### The optical coherence tomography (OCT) allows the detection of structural changes in the retina of TgCRND8 mice

Using the OCT-technique, we observed structural alterations of the retinal layers in male TgCRND8 mice at 4 months of age vs aged-matched WT mice.

**Figure 5:**
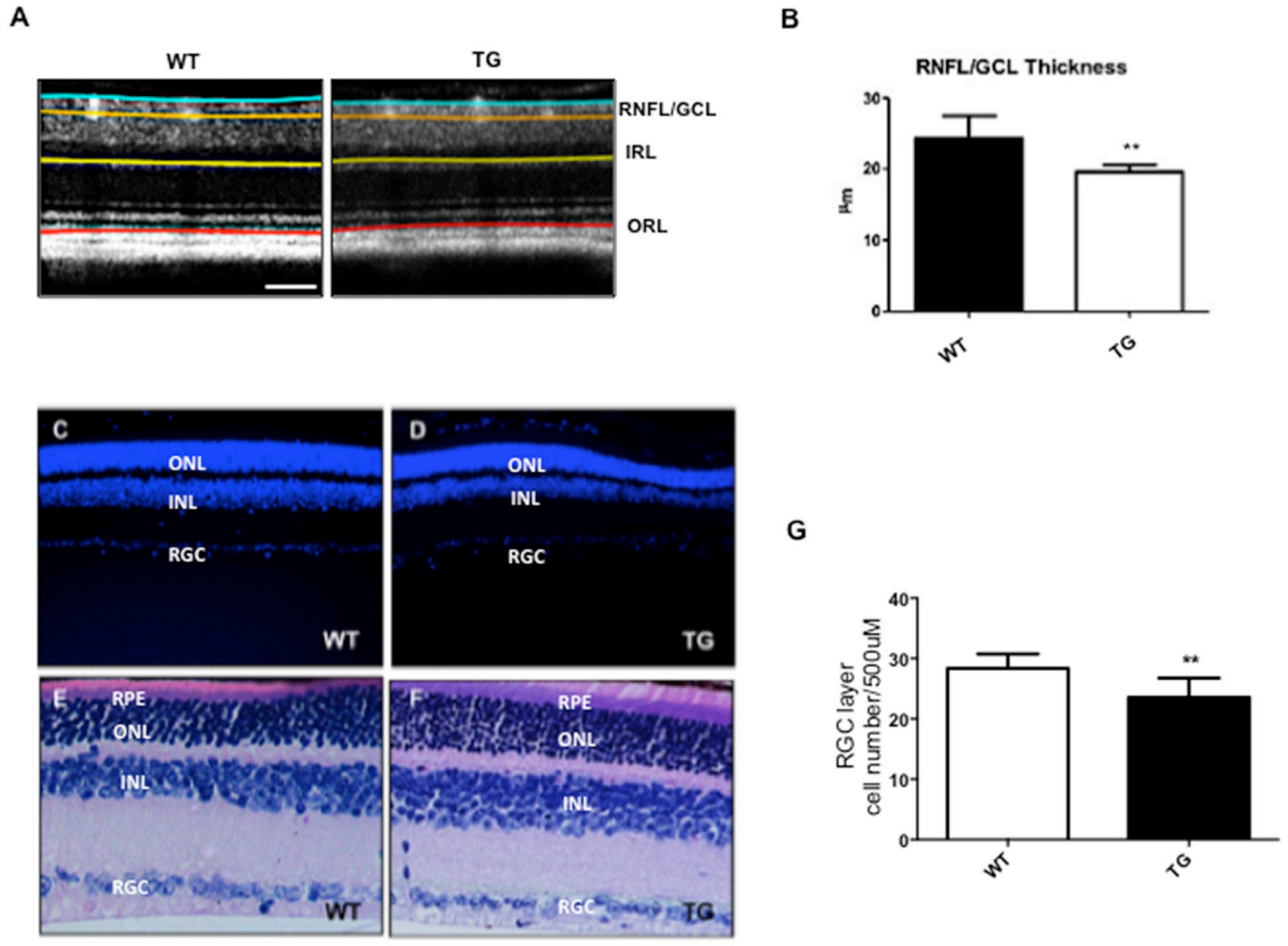
Reduced NFL/GCL thickness and RGC numbers in the retina of TgCRND8 mice The segmentation of retinal layers obtained by optical coherence tomography **(A)** and the relative quantification **(B)** showed a reduction of the NFL/GCL thickness in TgCRND8 mice [right] compared to WT [left]. Student’s t-test, ** p< 0.01. **(C-D)** Retinal sections from Tg [right] and WT animals [left] stained with DAPI [Scale 20 μm]. **(E-F)** WT [left] and TgCRND8 [right] mice depicting retinal morphological changes in H&E staining. **(G)** Quantification of the RGC cell number showed a reduction of RGC cells in TgCRND8 compared to WT mice. Data were expressed as mean ±SEM. Mann-Whitney test, ** p< 0.01, n=6.

We found a significant thinning of Retinal Nerve Fiber Layer\Ganglion Cell Layer (RNFL\GCL) (19.58±0.94 vs 24.29±3.14 mean (μm) ±SD; p< 0.01) in TgCRND8 compared to WT mice (Figure 5A-5B), whereas the thickness of all the other retinal layers remained equal.

We then verified if changes in the RNFL were due to loss of the RGCs, by performing DAPI staining (Figure 5C-5D) and Hematoxylin-Eosin histology (Figure 5E-5F) and counting the number of cells in the RGC layer (p< 0.01, Figure 5G). We found a 20% loss of RGCS in the retina of TgCRND8 mice, if compared to WT mice (Figure 5G) that can explain the significative thickness of the RNFL found (p< 0.01, Figure 5B). These results combined with previous evidence that AD markers strongly labeled the RGC layer (see Figure 1) supports the hypothesis that toxic species accumulate in the retina, and affect preferentially RGC, causing thinning of the RNFL.

### D-JNKI1 treatment prevents loss of RGC and structural changes in the RNFL in TgCRND8 mice

TgCRND8 mice were chronically treated with the specific JNK inhibitor peptide D-JNKI1 by intraperitoneally injection as previously described [24-25] for 4/5 months (starting at 4 until 8 months of age). At the end of the treatment their retinas as well as the whole eyes and optic nerves were analyzed. In the retina, we performed RGCs count to determine if D-JNKI1 treatment was able to prevent their death, while in the whole eyes we tested the effect of D-JNKI1 on the biochemical AD markers.

**Figure 6:**
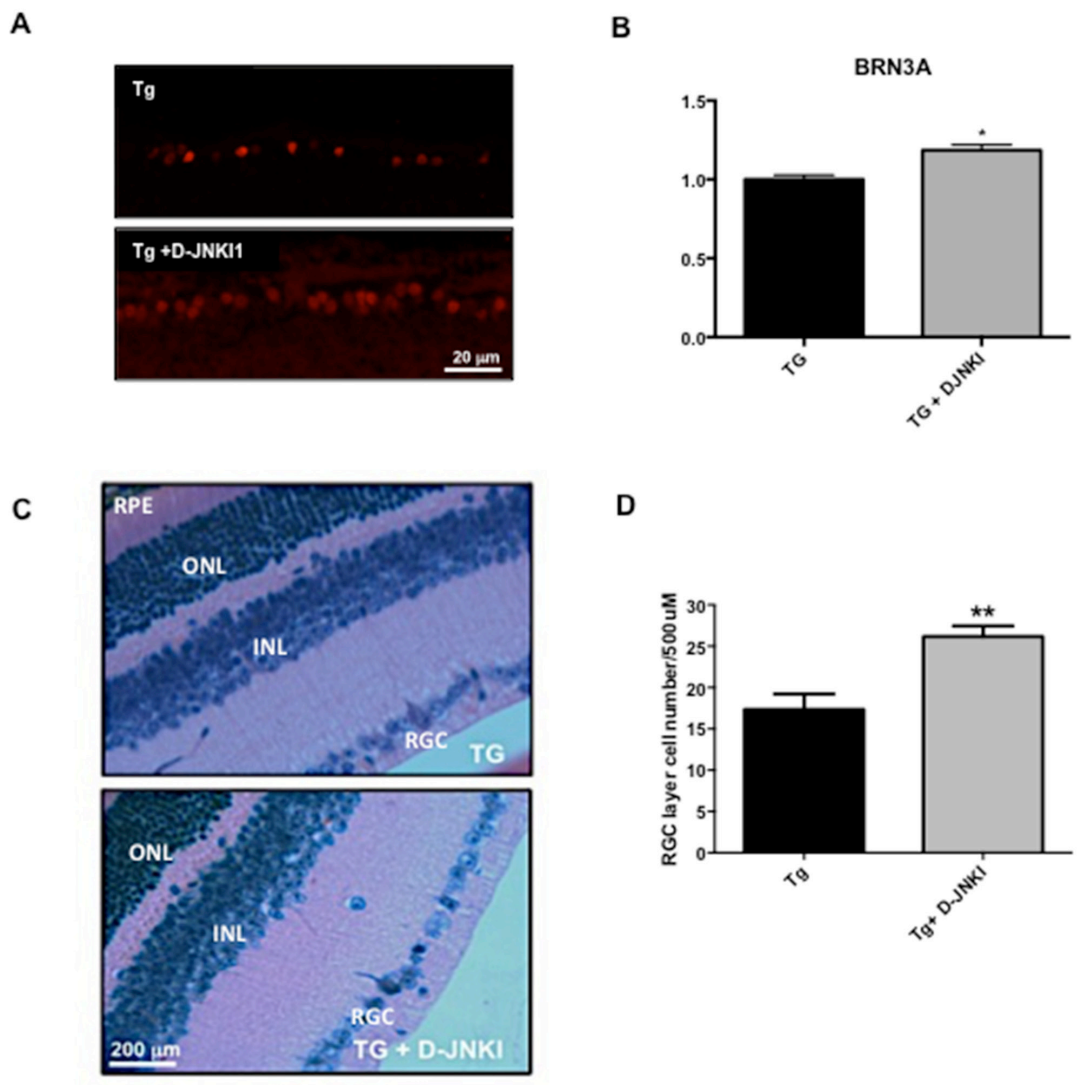
D-JNKI1 increases BRN3A and RGC numbers in retina of TgCRND8 mice **(A)** Immunostaing of retinal sections performed with BRN3A [a specific marker for RGC nuclei] and **(B)** relative quantification showed an increase of the number of BRN3A+ cells in D-JNKI1 treated Tg vs untreated tg animals. Scale 20 μm. Student’s T-test: *p< 0.05; n=5. **(C)** Retinal sections and **(D)** correlated RGC quantification from D-JNKI1 treated Tg [in the top] and untreated mice [in the bottom] stained with H&E staining [scale 200 μm]. Quantification of the RGC cell number showed an increase of RGC cells in D-JNKI1 treated TgCRND8 compared to untreated TgCRND8 mice. Data are expressed as mean ±SEM. Mann-Whitney test, ** p< 0.01 [n=6].

Retinal sections were stained for brain-specific homeobox/POU domain protein 3A (BRN3A, a specific marker for RGC nuclei), and positive nuclei were counted. BRN3A positive cells were 15% higher in D-JNKI1 treated TgCRND8 vs untreated TgCRND8 animals (23.40±5.15 vs 27.00±1.22, P=0,04; see Figure 6A-6B). This result was also confirmed by using the Hematoxylin and Eosin histology. We proved that the number of RGC was increased by 34% in D-JNKI1-treated vs untreated TgCRND8 mice (Figure 6C-6D), confirming the neuroprotective effect of D-JNKI1 against AD-related ocular neuropathology.

**Figure 7:**
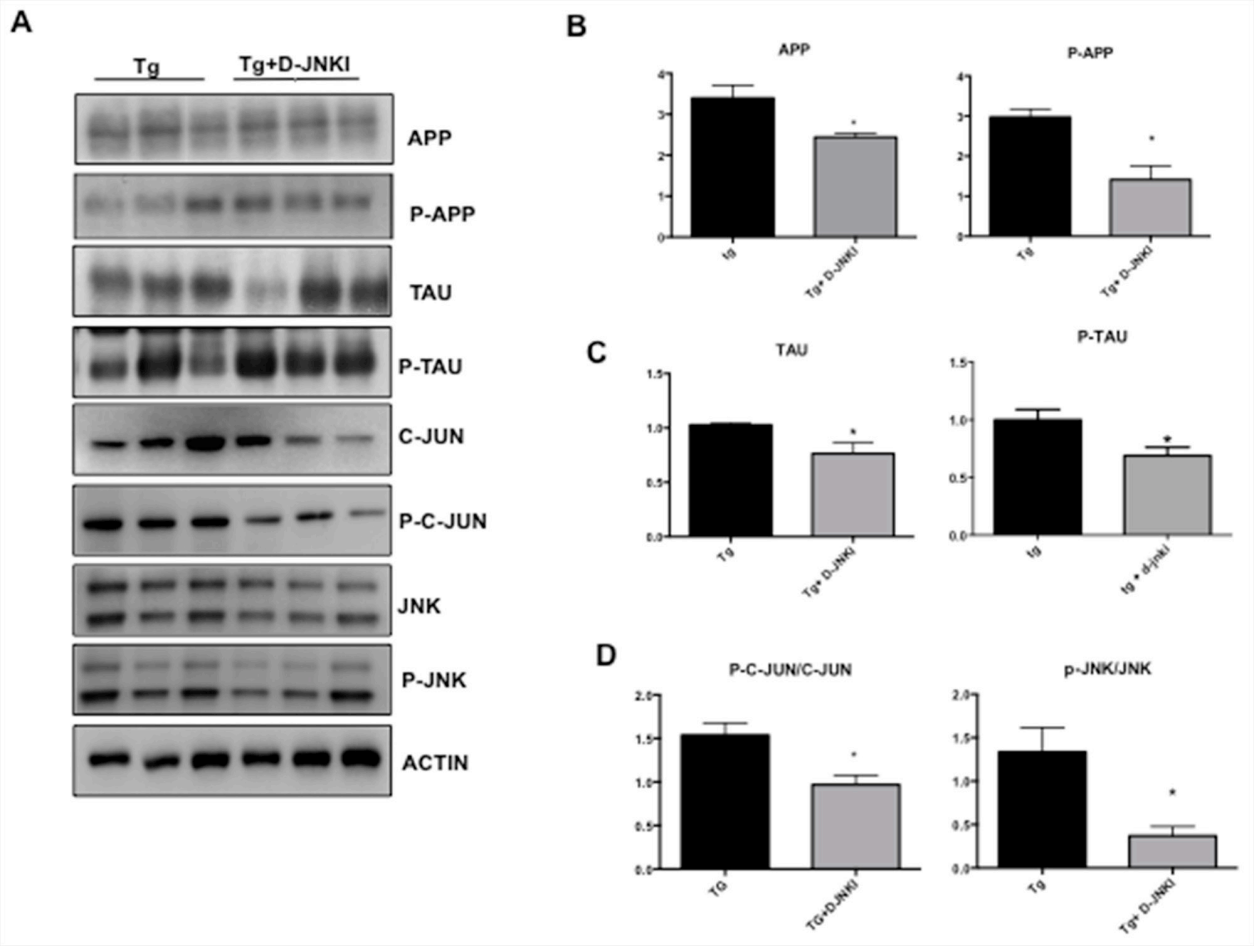
D-JNKI1 decreases APP and P-Tau levels in whole eye extract of TgCRND8 mice **(A)** Western blot analysis and relative quantification **(B)** showed a decrease of APP, P-APP, **(C)** Tau and P-Tau in whole extract of eye from D-JNKI1 treated Tg animals compared to untreated tg animals. Also P-c-Jun/c-Jun and P-JNK/JNK ratio **(D)** were decreased in D-JNKI1 treated Tg vs untreated Tg mice. Student’s T-test: *p< 0.05; n=6].

We then tested the effect of D-JNKI1 against AD hallmarks by Western blotting analysis in whole eye homogenates. In TgCRND8 mice chronically treated with D-JNKI1, we found a significant decrease in both total and phosphorylated levels of APP and Tau (p< 0.0001, Figure 7A-7B and Tau p< 0.0001, Figure 7A-7C). Furthermore, to investigate the D-JNKI1 impact on JNK pathway, we evaluated the P-c-Jun/c-Jun and p-JNK/JNK ratios in treated and untreated TgCRND8 and observed a significant decrease in the P-c-Jun/c-Jun ratio (equal to 40%) and p-JNK/JNK ratio (equal to 70%) (p< 0.05 Figure 7A-7D).

### D-JNKI1 treatment prevents the optic nerve degeneration in TgCRND8 mice

**Figure 8:**
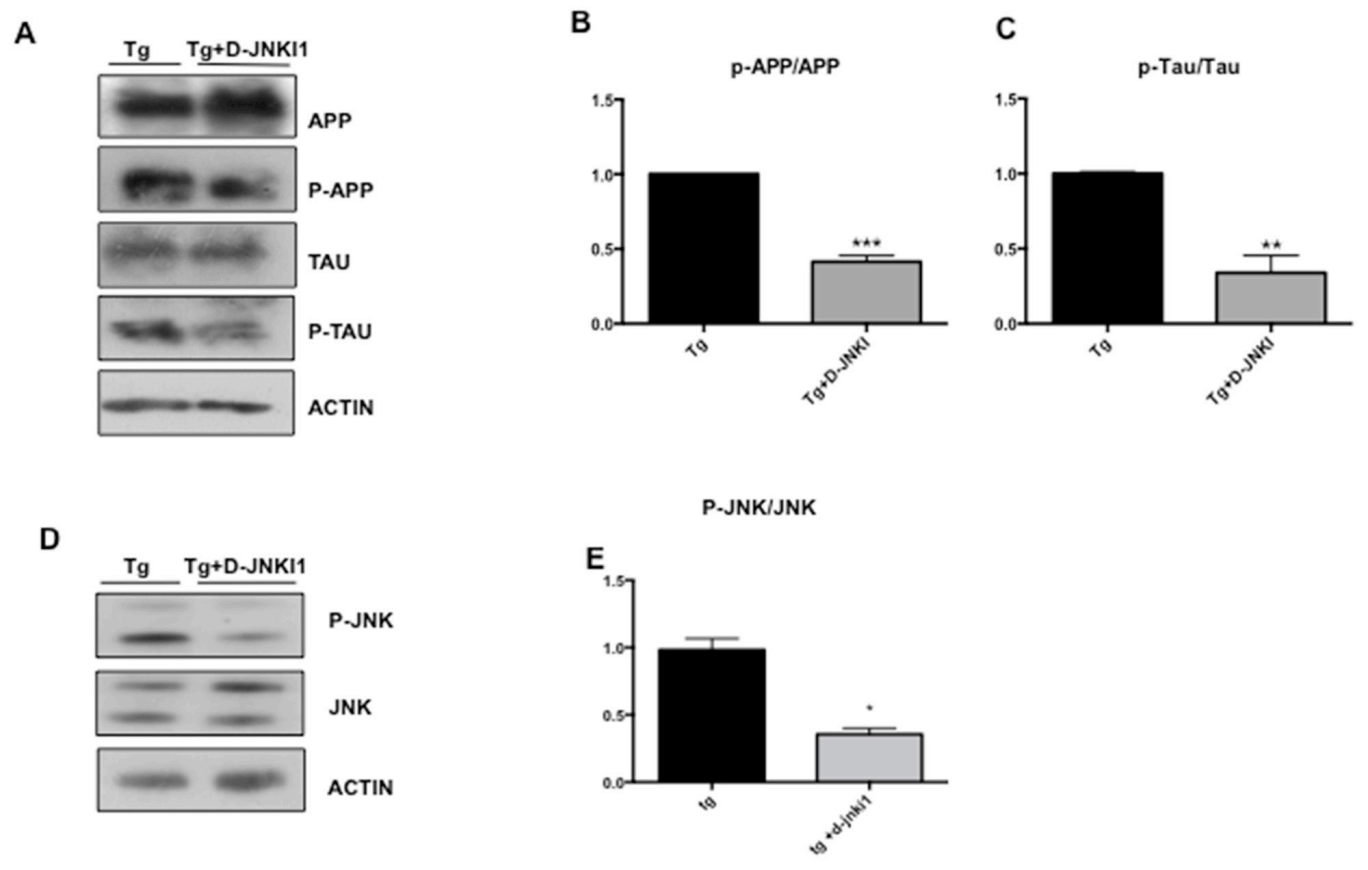
D-JNKI1 decreases P-APP/APP, P-Tau/Tau and P-JNK/JNK levels in the optic nerve of TgCRND8 mice **(A)** Western blot analysis and **(B-C)** relative quantification showed a decrease of P-APP/APP ratio and P-Tau/Tau ratio in the optic nerve from D-JNKI1 treated Tg animals compared to untreated tg animals. Student’s T-test: **p< 0.01; *** p< 0.001 n=5. **(D)** Western blot and **(E)** relative quantification showed a decrease of P-JNK/JNK ratio in the optic nerve of D-JNKI1 treated Tg compared to untreated tg animals. Data are expressed as mean ±SEM. Student’s T-test: *p< 0.05, *** p< 0.001 [n=6].

Finally, we verified if treatment with D-JNKI1 prevents the optic nerve degeneration observed in TgCRND8 mice. We performed Western blotting analysis on optic nerve homogenates, probing for p-APP/APP, p-Tau/Tau and p-JNK/JNK ratios in D-JNKI1 treated and untreated TgCRND8 mice. In accordance to the protective effect previously observed on RGCs, we found a significant decrease of p-APP/APP and p-Tau/Tau ratios in the optic nerve of D-JNKI1 treated *vs* untreated TgCRND8 mice (APP p< 0.001, Figure 8A-8B and Tau p< 0.01 Figure 8A-8C), due to inactivation of the JNK pathway. In fact, as expected, the level of p-JNK/JNK were significantly decreased in D-JNKI1 treated TgCRND8 mice vs untreated (Figure 8D-8E). These results confirmed the protective role of D-JNKI1 against AD pathological manifestations.

## DISCUSSION

Alzheimer disease (AD) is the most common form of dementia and to date no treatments are available to cure or slow AD progression. One explanation for the inefficiency of the therapeutic strategies is due to the fact that treatments start at later stages of AD, in the presence of irreversible brain damages (neuronal and synapse loss). Therefore, it is important to understand and decode the cellular and molecular mechanisms underlying AD pathogenesis as well as find new methods to monitor the beginning and the progression of the pathological process. Recently, many efforts have been done to obtain early detection methods for the diagnosis of AD. New approaches in prevention trials include positron emission tomography (PET) studies of glucose metabolism combined with genetic risk assessment as well as use of biomarkers, such as measurements of beta-amyloid and tau levels in the cerebrospinal fluid [28]. However, these methods have been reported to be too expensive or invasive. Several evidences in the literature describe that many patients affected by AD report visual impairment [29-31], suggesting a correlation between ocular and AD pathologies [32-35]. In particular, a recent study proved that retinal thickness is decreased in both AD and mild cognitive impairment (MCI) patients compared to controls [36]. Importantly, these ocular defects can manifest before the onset of dementia [8, 9] and can be detected at early stages of AD by high resolution imaging technologies, such as optical coherence tomography (OCT). OCT is a non-invasive technique able to discriminate among different cellular populations into the retina and to quantify thinning of the retinal nerve fiber layer (associate to the loss of intra-retinal RGC axons). For these reasons, visual pathological symptoms can be used for early diagnosis of AD, as well as for monitoring the progression of the disease. To date, little is known about the link between visual impairments and AD and the nature of the visual systems alterations. Moreover, it is not clear if accumulation of Aβ peptides and Tau may occur in the eye and may be responsible for the structural changes observed in AD patients. Finally, the molecular mechanisms leading to retina thinning in AD patients are poorly understood. Investigating the cellular and molecular mechanisms leading to visual impairment in AD patients may allow the identification of new therapeutic targets that can be used for the treatment of visual alterations as well as in a more general view of brain dysfunction.

Therefore, the goal of this study was to: i) verify if murine models of AD present similar structural alterations of the visual system, focusing on TgCRND8 mice, a well characterized model of AD [24-27, 37]; ii) detect eventual accumulation of Aβ peptides or Tau in the eye of TgCRND8 mice; iii) study the molecular mechanisms leading to structural changes in the retina, focusing on JNK signalling pathway; iv) test the protective role of D-JNKI1, a specific JNK inhibitors, in the eye.

We found that TgCRND8 mice show ocular alterations, similar to the ones found in AD patients [11, 38-39]. In fact, by using OCT, we observed in TgCRND8 mice a substantial thinning of the retinal nerve fiber layer (RNFL) as well and a significant reduction of the RGC number counted by classical histological and immunohistochemical methods. Moreover, our results confirm, as previously described in the literature [32, 40-47], that the reduction in the peri-papillary RNFL, detected by OCT, is due to RGC loss. To understand if the retinal degeneration in TgCRND8 mice mirrors AD-related changes observed in the brain parenchyma (in particular hippocampus and cortex), we investigated the presence of amyloid and Tau deposition in the retina of TgCRND8 mice. We found that APP and p-APP levels were increased in the retina of TgCRND8 mice, followed by accumulation of Aβ_1-42_ toxic species, as previously observed in the brain parenchyma [24]. We also found a significant P-Tau deposition in the retina of TgCRND8 animals by immunohistochemical and biochemical analyses, using three different specific antibodies to detect Tau protein and its hyperphosphorylated form. Consistent with these data, we found a similar increase in the level of APP, p-APP and p-Tau as well as accumulation of Aβ_1-42_ oligomers in total eye homogenates. Our data are in line with those of Koronyo-Hamaoui et al. [48] that detected amyloid plaques in the retinal of APP/PS1 transgenic mice, another murine model of AD mouse model.

As in the brain parenchyma, where the level of Aβ oligomers, P-APP and P-Tau correlates with the severity of synaptic loss and memory deficits, in the retina, accumulation of Aβ oligomers, P-APP and P-Tau specifically in the RGC layer leads to RGC degeneration and consequently to thinning of the RNFL at early stages of the disease.

To understand the molecular mechanisms correlating Aβ accumulation and death of RGC, we analysed the activation of the JNK signally pathway in the retina of TgCRND8 mice. Many reports, including our work on TgCRND8 mice, have been shown that JNK is a key stress modulator, that is activated by Aβ oligomers and initiates the degenerative mechanisms affecting neurons in the brain.

Moreover, JNK pathway contributes to AD progression, being the main kinase responsible for APP [24-25, 27, 49] and Tau phosphorylation [26], therefore promoting their accumulation in the brain. Here we found that similar mechanisms applied for the retina. In fact, we reported activation of the JNK pathway and its selective target c-Jun in the retina of TgCRND8 mice. Our data support the hypothesis that in the retina of AD patients, as well as in the brain, an abnormal accumulation of Aβ toxic species and P-Tau leads to activation of the JNK pathway, which increases the production of these toxic species and leads to neuronal degeneration.

To prove that JNK activation is responsible for the loss of RGC and the thinning of the RNFL, we treated TgCRND8 mice with D-JNKI1, a powerful and specific JNK inhibitor. D-JNKI1 prevented the structural changes observed in TgCRND8 mice, by inhibiting the phosphorylation of APP and Tau as well as the accumulation of Aβ oligomers in the retina. In support of our hypothesis, other studies have correlated JNK activation to RGC death in different models of retina degeneration, such as glaucoma, retina ischemia and excitotoxicity [50-53]. Moreover, the safety, tolerability, and the good systemic diffusion of D-JNKI1 in the treatment of post-surgery or post-trauma intraocular inflammation have been already described [54]. These results reinforced the idea that treatment with D-JNKI1 is an innovative and potentially neuroprotective strategy against different ocular pathologies, including the ones related to AD.

Importantly, systemic application of D-JNKI1 passed the first clinical trial phase and is well tolerated by patients; while it is in clinical trial phase III for the treatment of post-surgery or post-trauma intraocular inflammation [54].

In summary, our findings support the hypothesis that ocular pathological changes observed in AD models are predictive of the brain pathology. In fact, both ocular and brain neurodegeneration shared common pathological features (increased level of P-APP and P-Tau ad accumulation of Aβ oligomers), and degenerative mechanisms (JNK activation).

The availability of non-invasive imaging techniques (OCT), which are able to detect subtle, early changes at retinal level, opens a window for early detection of AD. As the retina cytology is much simple than that of the brain, this provided an excellent model to investigate the degenerative processes, signalling mechanisms, and to test novel neuroprotective agents. This similarity between the brain and the retina provides also an excellent experimental model for the examination of the efficacy of novel drugs as well as their pharmacokinetic and pharmacodynamics properties, offering several advantages in terms of cost, time, and analytical methods.

## MATERIALS AND METHODS

### Animals

In this study, we used TgCRND8 transgenic male mice. TgCRND8 mice expressed a mutant form of APP 695 containing both the Swedish (KM670/671NL) and Indiana (V717F) mutations on a hybrid C3H/B6 genetic background and exhibit extensive amyloid deposition by 3 months of age [37]. Age matched wild-type male mice (129 SV) served as controls. Mice from Jackson Laboratories, USA, were bred at IRCCS Mario Negri Institute of Pharmacological Research in a specific pathogen free (SPF) facility with a regular 12:12 h light/dark cycle (lights on 07:00 a.m.), at a constant room temperature of 22 ± 2°C, and relative humidity approximately 55 ± 10%. Animals were housed [4 per group] in standard mouse cages, all with (hard wood shavings) as bedding material, *ad libitum* food (Global Diet 2018S, Harlan Italy) and water. No environmental enrichment was used because it tends to improve the signs of AD pathology in mouse models [55-56]. Procedures involving animals and their care were in accordance with national and international laws and policies. The Mario Negri Institute for Pharmacological Research (IRCCS, Milan, Italy) Animal Care and Use Committee (IACUC) approved all protocols, which were conducted according to the institutional guidelines, in compliance with Italian laws.

### Animal treatment

All animals (Tg or WT) were intraperitoneally injected with vehicle (PBS) or D-JNKI1 (22 mg/kg) once a month from 4 to 8 months of age [57-58]. The weight of the animals was recorded before each treatment. Mice were treated always at the same time of the day (9:00–10:00 A.M.) in a specific room inside the animal facility, following a randomized order. Each single mouse was our experimental unit. The description of D-JNKI1 structure was reported by Davoli et al., 2014 [59].

### Optical coherence tomography (OCT)

*In vivo* analysis of the retina was performed using the Micron IV instrument combined with the Image-Guided 830nm OCT (Phoenix Research Laboratories, Pleasanton, CA, USA).

Anesthesia was induced by intraperitoneal injection of 80 mg/kg Ketamine, 10 mg/kg Xylazine (Sigma-Aldrich, Munich, Germany). To obtain mydriasis, a drop of tropicamide 0.5% (Visumidriatic, Tibilux Pharma, Italy) was instilled in each eye. The cornea was kept moist with an ophthalmic solution of hydroxyethylcellulose (Gel 4000 2%; Bruschettini, Italy). The OCT pictures were acquired with a bidimensional scan (B-scan), performing a circular scan of 550 μm of diameter around the optic nerve head. For all animals, both eyes were examined and the results were averaged.

The segmentation of retinal layers was performed using Insight software (Phoenix Research Laboratories), and four types of measurements were conducted:

1) Nerve Fiber Layer/ Ganglion cell Layer (NFL/GCL): from the inner limiting membrane to the limit between GCL and the Inner Plexiform Layer (IPL); 2) Inner Retinal Layers (IRL): from the IPL limit to the margin between the Inner Nuclear Layer (INL) and the Outer Plexiform Layer (OPL); 3) Outer Retinal Layers (ORL): from the INL/OPL margin to the external margin of the Outer Segment (OS) of the photoreceptors; 4) Total Retinal Thickness (RT): from the Nerve Fiber Layer/Ganglion cell Layer (NFL/GCL) to the external margin of the OS.

When performing OCT in mice it is quite difficult to discern between GCL and NFL [60]. To obviate to this problem these two layers were segmented together (GCL/NFL).

### Immunohistochemistry

After imaging, animals were sacrificed by cervical dislocation and eyes and optic nerves were harvested for light microscopic evaluation, fixed in 4% (w/v) PFA, processed in an automatic tissue processor [Leica], and embedded in paraffin. Eye tissues were mounted and subjected to hematoxylin and eosin staining (Sigma-Aldrich, see Fischer AH et al., 2008) [61]. Cell density in the GCL was determined for each eye by counting the number of cells in the middle part of retina over a distance of 300 μm (200 μm–500 μm from the edge of the optic disc). The thickness of inner plexiform layer was also evaluated and compared between WT and TGCRND8 mice.

To detect the typical hallmarks of AD as Aβ plaques, hyperphosphorylated Tau and APP we used different primary antibodies: 6E10 (1:500, Covance, Emeryville, CA, USA), APP (cat. #2024170, 1:250, Millipore, Billerica, MA, USA) and AT100 (cat. #MN1060, 1:500, Euroclone). After deparaffinization, eyeballs sections (3 μm thick; four slices per mouse) were unmasked with sodium citratePH 6 in the microwave for 2 minutes incubated for 1 h at room temperature with blocking solutions (6E10: 10% normal goat serum (NGS) plus 0.3% Triton X-100; APP: 0.5% Triton X-100 plus 10% NGS; AT100: 0.3% Triton X-100 plus 10% NGS) and then overnight at 4°C with the primary antibodies. After incubation with the biotinylated secondary antibody (1:200; 1 h at room temperature; Vector Laboratories, Burlingame, CA, USA), the sections were incubated for 30 minutes at room temperature with the avidin-biotin-peroxidase complex (Vector Laboratories) and diaminobenzidine (Sigma-Aldrich, Italy). Tissue analysis and image acquisition were done using an Olympus image analyzer and the Cell-R software.

### Immunofluorescence analysis

After the sacrifice of the animals, the eyes were fixed in 4% PFA for 1h and then rinsed in PBS. Cornea and lens were removed and the retina, attached to the sclera, was incubated overnight in 30% sucrose before being embedded within optimal cutting temperature compound (Sigma, St. Louis, MO, USA).

Eyes were sectioned at a thickness of 12 μm and sections containing the optic disk were utilized for subsequent analysis. Sections were permeabilized with 0.3%Triton X-100 and 2% BSA in PBS for 30 minutes at room temperature and blocked with 10% donkey serum for 30 minutes at room temperature. The primary antibody anti POU4F1/BRN3a (C-20) (sc-31984, Santa Cruz Biotechnology, CA, USA), was added and incubated overnight at 4°C (dilution 1:100). The following day, retinal sections were incubated with TRITC conjugated secondary antibodies (Jackson Immunoresearch, West Grove, PA, USA) diluted 1:50 in PBS for 1h. Nuclear staining was performed with DAPI (Sigma). Slides were mounted with Vectorshield medium (Vector Laboratories Inc, Burlingame, CA, USA). Omission of primary antibody was used as a staining control.

Positive cells were counted over a distance of 300 μm (250 μm–550 μm) from the center of the optic nerve head as previously described [62]. Four consecutive sections were analyzed. Images were acquired by epifluorescence microscopy using an Axio Plan 2 microscope and an MRc camera (Carl Zeiss, Jena, Germany). Identical exposure time was used for all the samples. Images measures and analysis were performed by ImageJ software.

### Western blot

Protein concentrations were quantified using the Bradford Assay (Bio-Rad Protein Assay 500-0006, Munchen, Germany) 5 μg of TIF extracted proteins were separated by 10% SDS polyacrylamide gel electrophoresis. PVDF membranes were blocked in Tris-buffered saline (5% non fat milk powder, 0.1% Tween20, 1 h, room temperature). Primary antibodies were diluted in the same buffer (incubation overnight, 4 °C) using: APP (cat. #2024170, 1:1000, Millipore, Billerica, MA, USA), p-APP (cat. #MABN10, 1:1000, Millipore, Billerica, MA, USA), anti Tau-5 (cat. #MABN162, 1:1000, Millipore Mab 361), p-Tau (cat. #MABN388, 1:1000, Millipore, Billerica, MA, USA), c-Jun (cat. #9165, 1:1000, Cell Signaling Technology, Danvers, MA, USA), p-c-Jun [Ser63] (cat. #9164, 1:1000, Cell Signaling Technology, Danvers, MA, USA),p-JNK (cat. #9251, 1 : 1000, Cell Signaling Technology, Danvers, MA, USA), JNK (cat. #9252, 1 : 1000, Cell Signaling Technology), anti-Actin (cat. #MAB1501, 1:5000, Millipore, Billerica, MA, USA) and at least six independent experiments were performed. Blots were developed using horseradish peroxidase-conjugated secondary antibodies (Santa Cruz Biotechnology) and the ECL chemiluminescence system (Promega). Western blots were quantified by densitometry using ImageQuant TL software (Amersham Biosciences, Amersham, UK) and was based on at least three independent experiments.

### Quantification of Aβ _1-40_ and Aβ _1-42_

Eye balls and retinas from TG and WT mice were homogenized in a Tris buffer containing 50 mm Tris-HCl, ph 7.4, 150 mM NaCl, 50 mM EDTA, 1% Triton X-100, and 2% protease inhibitor. After centrifugation (15,000 rpm, 21,000 × *g*, 4°C for 25 minutes), the supernatant was retained as the Triton-soluble fraction (soluble Aβ). The pellet was homogenized for a second time in the presence of 70% formic acid (FA) (10% v/w) and ultracentrifuged [55,000 rpm, 100,000 × *g*, 4°C, 1 h], and the resulting FA-extracted supernatant was neutralized with 1 M Tris buffer, pH 11, representing the FA-extracted insoluble fraction. Levels of Aβ_1–40_ and Aβ_1–42_ in each fraction were quantified by sandwich ELISA (IBL ELISA kit nr RE59721, RE59751).

### Statistical analysis

Statistical analysis was done using Graph Pad Prism 6 program. WB data, ELISA data and neuronal counts were analyzed using two-way ANOVA, followed by Tukey’s *post hoc* test. All data were expressed as mean ± SEM with statistical significance given at p< 0.05.

## Abbreviations

AD: Alzheimer’s disease
APP: amyloid precursor protein
CNS: central nervous system
NFTs: neurofibrillary tangles
JNK: c-Jun N-terminal kinase
INL: inner nuclear layer
IPL: inner plexiform layer
IRL: inner retinal layers
OCT: optical coherence tomography
ONL: outer nuclear layer
OPL: outer plexiform layer
ORL: outer retinal layers
RGC: retinal ganglion cell
RNFL: retinal nerve fibers layer
